# Long-term impacts of prenatal synthetic glucocorticoids exposure on functional brain correlates of cognitive monitoring in adolescence

**DOI:** 10.1038/s41598-018-26067-3

**Published:** 2018-05-16

**Authors:** Liesa Ilg, Manousos Klados, Nina Alexander, Clemens Kirschbaum, Shu-Chen Li

**Affiliations:** 1 Chair for Lifespan Developmental Neuroscience, Faculty of Psychology, Technische Universität Dresden, Zellescher Weg 17, 01062 Dresden, Germany; 2 Department of Biomedical Engineering, Aston University, MB555 Aston Triangle, Birmingham, B47ET UK; 3 Chair for Biopsychology, Faculty of Psychology, Technische Universität Dresden, Zellescher Weg 19, 01602 Dresden, Germany; 4 Department of Psychology, Faculty of Human Sciences, Medical School Hamburg, Am Kaiserkai 1, 20457 Hamburg, Germany

## Abstract

The fetus is highly responsive to the level of glucocorticoids in the gestational environment. Perturbing glucocorticoids during fetal development could yield long-term consequences. Extending prior research about effects of prenatally exposed synthetic glucocorticoids (sGC) on brain structural development during childhood, we investigated functional brain correlates of cognitive conflict monitoring in term-born adolescents, who were prenatally exposed to sGC. Relative to the comparison group, behavioral response consistency (indexed by lower reaction time variability) and a brain correlate of conflict monitoring (the N2 event-related potential) were reduced in the sGC exposed group. Relatedly, source localization analyses showed that activations in the fronto-parietal network, most notably in the cingulate cortex and precuneus, were also attenuated in these adolescents. These regions are known to subserve conflict detection and response inhibition as well as top-down regulation of stress responses. Moreover, source activation in the anterior cingulate cortex correlated negatively with reaction time variability, whereas activation in the precuneus correlated positively with salivary cortisol reactivity to social stress in the sGC exposed group. Taken together, findings of this study indicate that prenatal exposure to sGC yields lasting impacts on the development of fronto-parietal brain functions during adolescence, affecting multiple facets of adaptive cognitive and behavioral control.

## Introduction

During gestation, the fetus is highly responsive to steroid hormones, particularly the glucocorticoids. The level of endogenous glucocorticoids is tightly regulated prenatally and its rapid increase towards the end of gestation is crucial not only for lung maturation but also for various aspects of normative neurodevelopment. Glucocorticoids initiate the terminal maturation of neurons, modulate the growth of axons, dendrites and synapses, as well as affect cell survival^1^ (see also a recent review^2^ of *in-vitro* evidence for effects of steroid hormones in animal models and human neuronal tissues). Furthermore, evidence from available *in-vivo* human structural brain imaging studies shows that maternal cortisol levels at different stages of prenatal development yield different impacts on brain development during childhood. Higher levels of maternal cortisol during early gestation were found to yield long-term consequences on female children’s amygdala volume and problems in affective behavior during childhood^3^, whereas higher levels of maternal cortisol during 3^rd^ trimester have been associated with greater cortical thickness in frontal brain regions and better cognitive performance in children^4^. Although moderate influences through maternal cortisol during late gestation could have beneficial effects^4^, overexposures to stress hormones are risk factors for prenatal development (see^5^ for review), with lasting effects that include dysregulated hypothalamic-pituitary-adrenal (HPA) axis^6^, altered structural brain development^3^ as well as affective and behavioral problems^3,7,8^ in later life periods.

During non-normative development associated with the risk of preterm birth, the levels of glucocorticoids during prenatal development can also be affected through exposures to synthetic glucocorticoids (sGC), besides the influences of maternal cortisol. About 10% of all pregnancies are in threat of premature birth and about 70% to 90% of women at risk for preterm delivery are commonly given sGC in the form of betamethasone or dexamethasone to promote fetal lung maturation^9–11^. Whereas maternal cortisol is mainly converted into inactive cortisone, the sGC readily crosses the placenta^12^, hence can potentially expose the fetus to supraphysiological levels of glucocorticoids that may negatively affect development. Animal studies have revealed that prenatal sGC exposures perturb brain development in regions targeted by the glucocorticoid receptors, notably the medial prefrontal cortex, the anterior cingulate cortex (ACC) and hippocampus^13–15^. Although there is some evidence indicating that prenatal exposure to sGC treatment is associated with increased risk for later mental health problems in childhood and adolescence^16^, human studies about effects of prenatal sGC exposure on brain development are very limited and focus mainly on structural brain development. An early brain imaging study showed that term-born infants exposed to multiple doses of prenatal sGC treatment showed reduced cortical folding^17^. Of note, a recent study reported greater cortical thinning in rostral ACC in term-born 6- to 10-year-old children who were exposed to sGC prenatally^18^. Moreover, the cortical thinning was related with behavioral affective problems, presumably reflects attenuated frontal behavioral control^18^. Going beyond brain structural development in childhood, here we investigated whether prenatal exposure to a single course of sGC around the 3rd trimester may yield lasting effects on the functional development of the fronto-parietal network, which underlie cognitive and behavioral control, during adolescence.

Thus far, human studies about persisting impacts of perturbed prenatal stress hormone levels on brain structures^3,4,17,18^ or affective processes^3,6,7^ examined effects that lasted until childhood. The question of whether adolescent brain functional development may also be affected by prenatal sGC exposures is still open. Longitudinal research on structural brain development has revealed that a few brain regions (e.g., the prefrontal cortex and hippocampus) interacting with the HPA-axis continue to mature in adolescence. In particular, the human medial prefrontal cortex shows a prolonged course of maturation that lasts well after puberty^19^; thus, lasting impacts of prenatal sGC exposure on functions subserved by the fronto-parietal network seem likely. Various sub-regions in the medial prefrontal cortex are known to play central roles in cognitive control and the monitoring of processing conflicts between competing stimulus features or action plans. Such monitoring processes are fundamental for adaptive cognitive and behavioral control by detecting conflicts and signaling the need for enhanced top-down attention and regulation^20–22^. Psychophysiological studies revealed that a brain event-related potential (ERP) associated with conflict monitoring is reflected by a negative deflection peaking around 200–300 ms after stimulus onset, with a fronto-central scalp distribution that is commonly known as the N2 component^20^. Previously we have shown that this component continues to mature in adolescence^23^. We thus investigated potential lasting impacts of prenatal sGC exposure on the N2 component during conflict monitoring and the associated source activations in the fronto-parietal network in teenagers.

We re-recruited participants of a prior child developmental sample in which effects of prenatal sGC on salivary cortisol stress reactivity^6^ and intelligence^24^ were studied. These term-born teenagers were between 14 to 18 years of age (mean age 16) at the time of our data collection. Most of them were attending gymnasium, which is a secondary school type after finishing primary school and represents the highest level of school education in Germany. The gymnasium school type generally prepares the adolescents for meeting the matriculation standard for further academic education at the university level (see Table 1 for sample characteristics and Methods presented after the main text for details about sample recruitment). Our sample included only participants who were born full term (cf.^25^), in order to exclude potential confounds related to preterm delivery (e.g., lower birth weights or other birth outcomes affecting health status of the newborns) that may also contribute to neurobehavioral problems in later life periods^26^. The final sample included a group of teenagers (n = 28) whose mothers experienced problematic pregnancies and were treated with the standard single course of sGCs therapy (i.e., the PP/sGC group), as well as a comparison group of teenagers (n = 24) whose mothers underwent usual pregnancies without complications nor sGC treatment. The ERPs were recorded while the teenagers performed a modified version of the Cued Continuous Performance Task (CPT; see Methods presented after main text for details about the task), which effectively elicits stimulus-response conflicts^22,23^ when a prevalent response has to be suppressed (NoGo trials), compared to situations in which the dominant response is executed (Go trials).

**Table 1:** Demographic characteristics, basic cognitive abilities and Go-NoGo performance by group.

	PP/sGC	Comparison	Statistics
***Sample characteristics***			
*N*	28	24	
Age	16.25(1.2)	16.42(1.0)	*U* = *316*.*5(P* = *0*.*71)*
Gender (%male)	67.86	54.17	*X*^2^ = *0*.*53(P* = *0*.*47)*
Birth weight (g)	3080.00 (549.48)	3357.08 (494.29)	*t* = *−1*.*91(P* = *0*.*06)*
Gestation at delivery	38.5(1.3)	39.2(1.2)	*U* = *199*.*5(P* = *0*.*03)*
APGAR score	9.25(0.64)	9.5(0.51)	*U* = 2*70*.*0(P* = *0*.*17)*
Tanner stage	4.58(0.7)	4.50(0.5)	*U* = 371(*P* = 0.93)
School Type (%Gymnasium)	60.7	62.5	*X*^*2*^ = *3*.*34(P* = *0*.*50)*
***Basic cognitive abilities***			
IPT RT(ms)	2064(296)	2039(260)	*t* = 0.19(*P* = 0.85)
SaW	13.2 (4.7)	14.7 (5.9)	*t* = 1.0*(P* = 0.32)
MSIT	1.61(0.13)	1.59 (0.11)	*t* = −0.72*(P* = 0.47)
***Performance in Go-NoGo task***			
Misses (%)	3.43(3.34)	3.41(3.29)	*U* = *341*.*5(P* = *0*.*9)*
RE error (%)	5.99(5.38)	4.94(4.30)	*U* = *298(P* = *0*.*49)*
PR errors (%)	7.42(7.30)	8.08(4.74)	*U* = 407.5*(P* = 0.19)
Go-RT (ms)	387(45)	374(54)	*U* = 282(*P* = 0.33)
Go-RTSD (ms) block 1	104.6(56)	104(65)	*U* = *308(P* = *0*.*62)*
Go-RTSD (ms) block 2	105(40)	101(52)	*U* = *319(P* = *0*.*76)*
Go-RTSD (ms) block 3	114(59)	103(59)	*U* = *289(P* = *0*.*40)*
Go-RTSD (ms) block 4	112(53)	108(57)	*U* = *301(P* = *0*.*53)*
Go-RTSD (ms) block 5	122(53)	94(38)	*t* = 2.17*(P* = 0.03)

*Note*: S.*D*. *in parenthesis;* If data violated the normality assumption, Mann-Whitney-U test (*U*) for non-normally distributed data was used; APGAR is a measure of the health of the newborn that assesses Appearance, Pulse, Grimace, Activity, and Respiration. The distribution of attended school type is indicated by the relative percentage of participants attending the Gymnasium (% Gymnasium). IPT RT = reaction time in Identical Pictures Task; SaW = correct reports in Spot-a-Word task; MSIT = cognitive interference measured by multisource interference task; RE error = response based errors/ PR = prime based errors/ RT = reaction time/ RTSD = intra-individual standard deviation of reaction time in CPT task.

In light of previous animal^13^ and human^18^ evidence, which suggest that prenatal sGC exposure affects, respectively, neuronal morphology and cortical thinning in the ACC, we expected altered amplitudes of the NoGo-N2 component in teenagers prenatally exposed to sGCs relative to the comparison group. Furthermore, based on studies consistently confirming neuronal generators of the error- or conflict-associated N2 component to be in the medial prefrontal regions involving the ACC (see^20,21^ for reviews) in children and adolescents^27,28^, we conducted cortical source localization analyses (see Methods for details about the analysis protocols) to examine potential effects of prenatal sGC exposure on source activations in the fronto-parietal network.

## Results

### Comparable sample characteristics between groups

Teenagers in the PP/sGC and comparison groups did not differ with respect to age, sex distribution, birth weight, the APGAR score measuring the health of newborns^29^, or the distribution of attended school types (e.g., gymnasium vs. other school types). We also assessed physical pubertal status measured by the Tanner scale^30,31^. Participants of this study were all in an advanced pubertal stage with the average Tanner stage scores around 4.6. The Tanner score did not differ between groups. The groups also did not differ in any of the basic cognitive abilities^32,33^ (see details in Table 1 and Methods at the end of the main text) that were assessed as control measures. Sex did not reveal any significant main or interaction effects with respect to the main behavioral and brain measures observed here (*P* > 0.1), and was thus not considered as a factor in further analyses. Furthermore, although on average the gestation at delivery was 0.7 week earlier in the PP/sGC group than the comparison group (*P* = *0*.*03*), including this variable or/and birth weight as covariate(s) into the statistical models for analyzing effects of group or group × condition interactions in main outcome measures (e.g., brain correlates of performance monitoring) did not affect the patterns of results (*p* values of group main effects remain at the level of *p* < 0.05 and the values of the group × condition effects remain at the level of *p* < 0.001); thus these variables were also not considered in further analyses.

### Behavioral performance assessed by the CPT task

Indices of performance accuracy and mean reaction time (RT) of the Go-NoGo CPT task did not differ between groups. The two groups were similar in their speed of responding during Go trials and committed equal amounts of all three error types (misses, response-based and prime-based errors) during NoGo trials (Table 1 shows values and statistics). We further tested the group effect with respect to intraindividual RT variability (indexed by within-person RT standard deviation across trials). The intraindividual RT variability in perceptual decision tasks has been established as an indicator for individual differences in the consistency of neurocognitive processes that correlate with dopamine modulation^34^ and reflects cognitive developmental status^32^. Furthermore, RT variability in later portion of cognitive tasks when information processing becomes slower and less consistent^35^ are known to reflect individual differences in lapses of sustained attention^35–37^. Of particular relevance, a previous study of adolescents with attentional deficit hyperactivity disorder^38^ showed that a higher level of prenatal maternal anxiety was associated with increased RT variability, specifically in the last block of a variant of the CPT task similar to that used in the current study. We thus also explored the potential group effect on RT variability during Go trials in each experimental block. The RT variability showed a slight increasing trend across blocks in the PP/sGC group, whereas this was not the case in the comparison group. In line with the previous finding^38^, only in the last experimental (5^th^) block the intraindividual RT variability (indicating lower response consistency) of the PP/sGC group was significantly higher relative to the comparison group (*P* = 0.03, see Table 1). This suggests that prenatal sGC exposure seems to attenuate processing consistency towards the end of the task when sustained attention was more in demand.

### NoGo-N2 effect at the scalp level

**Figure 1:**
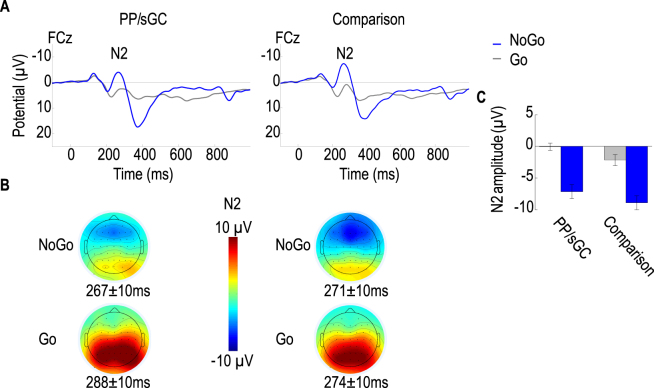
(**A**) Grand average ERPs at the channel FCz for Go and NoGo condition in the PP/sGC and comparison groups. (**B**) Topographical maps ± 10 ms around respective peak latency showing a fronto-central distribution of NoGo-N2 amplitudes in the PP/sGC and comparison groups. (**C**) Mean N2 amplitudes displayed for each condition and group (error bars indicate ± 1 standard error of the mean).

Regarding brain ERPs assessed at the scalp level, in both groups the Go and NoGo trials elicited a negative deflection around 280 ms after stimulus onset (Fig. 1A). Their topographical maps confirmed the typical fronto-central distribution of the N2 component (Fig. 1B). In line with previous findings of brain event-related potentials of error- or conflict-related monitoring^20,23^, a significant main effect of Condition (*F*(1,150) = 292.49, *P* < 0.001, $\eta$^2^ = 0.36) confirmed the expected increase of N2 amplitudes following NoGo trials relative to Go trials. The effect for Channel was also significant (*F*(1,150) = 22.64, *P* < 0.001, $\eta$^2^ = 0.04), reflecting larger negative deflections at the frontal (Fz) than fronto-central (FCz) electrode. Of particular interest were the significant main effects of Group (*F*(1,50) = 5.26, *P* = 0.03, $\eta$^2^ = 0.07) and the Condition × Group interaction (*F*(1,150) = 11.95, *P* < 0.001, $\eta$^2^ = 0.02). As shown in Fig. 1C, follow-up independent samples *t* tests, performed separately for the Go and NoGo condition, showed a statistically smaller NoGo-N2 amplitude in the PP/sGC than in the comparison group (*t* = 2.65, *P* = 0.01); whereas the Go-N2 amplitudes did not differ between the groups (*t* = 1.08, *P* = 0.28). These results indicate that teenagers in the PP/sGC group perceived (monitored) less stimulus-response conflict in NoGo trials relative to the comparison group.

### Condition effects at the source level

**Figure 2:**
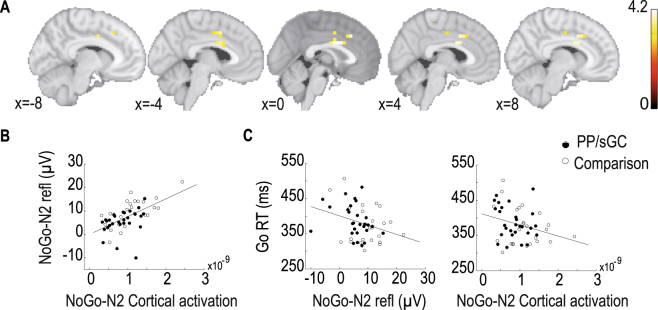
(**A**) Significantly stronger source activations for the NoGo relative to the Go condition across both groups (presented are slices of images around the midline showing relevant regions in the cingulate cortex and middle frontal gyrus). (**B**) Scatterplot showing the correlation between NoGo-N2 related cortical activation and NoGo-N2 amplitudes (reflected values) at FCz across both groups. (**C**) Scatterplots showing the correlation between RT of Go trials and, respectively, NoGo-N2 amplitudes (reflected values) at FCz (left) as well as NoGo-N2 related source activations (right).

Cortical activations were analyzed in the time window (250–280 ms) of the N2 components (see Methods for details of the analyses). As expected, various clusters of source activations in the fronto-parietal network were statistically stronger in the NoGo relative to the Go condition (*P* < 0.0001). Most of these effects are in regions known to implicate cognitive and behavioral control (Fig. 2A): namely, the cingulate cortex including the ACC (brodmann areas BA24, BA32), frontal lobe regions including the superior frontal gyrus, medial frontal gyrus (BA9), and the supplementary motor area (SMA). Moreover, source activations in these regions correlated positively with the NoGo-N2 amplitudes measured at channel FCz across groups (*r* = 0.58, *P* < 0.001; Fig. 2B). The relation also holds in each of the two groups (*r* = 0.69, *p* < 0.001 in comparison group; *r* = 0.31, *P* = 0.03, one-tailed in PP/sGC group). We further correlated NoGo-N2 amplitudes and the related cortical source activations with behavior performance across groups, while controlling for the effect of the group factor using Spearman’s rho for non-parametric correlation. The results (Fig. 2C) revealed negative correlations between RT of Go trials and the reflected NoGo-N2 amplitude (*rho* = −0.24, *P* = 0.04, one-tailed) or the associated cortical activations at the source level (*rho* = −0.23, *P* = 0.05, one-tailed). These findings indicate that teenagers with smaller N2 amplitudes and lower cortical source activations required more time to react to the target stimuli.

### Group effects at the source level

**Figure 3:**
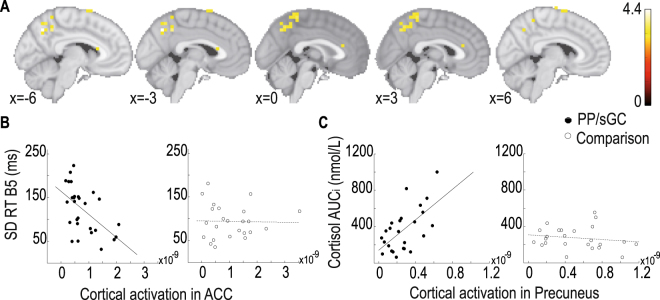
(**A**) Regions of significantly higher activations in the comparison group relative to the PP/sGC group during the NoGo condition (presented are slices of images around the midline showing relevant regions in the ACC, SMA and precuneus). (**B**) Scatterplots showing the correlation between source activation in the ACC and intraindividual RT variability in the sGC exposed and comparison groups. (**C**) Scatterplots showing the correlation between source activation in the precuneus and salivary cortisol reactivity to social stress in the sGC exposed and comparison groups.

We next tested for group effects on source activations associated with the NoGo-N2 component. Confirming effects observed at the scalp level, relative to the comparison group we found significantly lower activations (*P* < 0.0001) in the PP/sGC group in several regions recruited during NoGo trials, including: ACC, middle frontal gyrus, and the SMA. Moreover, though not revealed in the condition-based contrast, the activations in precuneus (BA7), cuneus (BA17) and temporal (BA21) regions were also significantly attenuated in the PP/sGC group (Fig. 3A). We further examined the relations between source activations in the ACC and behavioral response consistency during the CPT task, which was attenuated in prenatally sGC exposed teenagers. Furthermore, since heightened functional connectivity between precuneus and amygdala following acute social stress have been found to reflect individual differences in stress responses^39,40^, we examined the correlation between source activation in precuneus and salivary cortisol reactivity to social stress (see Methods for details about measuring salivary cortisol reactivity). In the PP/sGC group, source activation in the ACC correlated negatively with reaction time variability during the Go trials (*r* = −0.55, *P* = 0.002; Fig. 3B), whereas activation in the precuneus correlated positively with salivary cortisol reactivity to social stress assessed in a separate behavioral session (*r* = 0.61, *P* = 0.001; Fig. 3C). In the comparison group, no such relations were found. Together, these results indicate that although cortical activations in these regions were lower in the PP/sGC group than in the comparison group, the individual differences in behavioral response consistency and cortisol reactivity to social stress in prenatally sGC exposed adolescents reflect, respectively, individual differences in the functional recruitments of the ACC and precuneus.

## Discussions

Extending prior human studies on effects of prenatal maternal cortisol^3,4^ and sGC exposure^18^ on brain structural development during childhood, the present study investigated long-term persisting effects of prenatal sGC exposure on functional brain development in adolescence, focusing on regions in the fronto-parietal network that are crucially involved in cognitive conflict monitoring^20–23^ and stress regulation^39,40^. Assessing functional brain correlates of adaptive control using an established stimulus-response conflict monitoring task^22,23^, indeed we found that term-born adolescents whose mothers were treated with the prenatal sGC therapy in the 3^rd^ trimester showed attenuated behavioral response consistency during cognitive conflict monitoring, particularly towards the end of the task when sustained attention were particularly required. This behavioral effect is paralleled by their reduced amplitudes of the N2 component during NoGo trials (Fig. 1C). Furthermore, results of source localization analyses also revealed effects of prental sGC on functional activations in the fronto-parietal network, particularly in regions implicating adaptive cognitive and behavioral control (Fig. 3A).

### sGC exposure affects functional development in the fronto-parietal network

Activations in regions of the cingulate cortex have been established as the neuronal generators of the N2 component during conflict monitoring in many EEG source localization studies^20,27,28^. The present study showed that activations in various regions in the fronto-parietal network during conflict monitoring were reduced in adolescents prenatally exposed to sGC. Most notably, activations in the cingulate cortex, middle frontal gyrus, SMA as well as precuneus in the parietal cortex were lower in a contrast comparing them with teenagers in the comparison group (Fig. 3). These findings are in line with an earlier result showing prenatal sGC exposure altered cortical thickness of ACC in children^18^. Relations between cortical thickness and cognitive development in preterm-born adolescents and young adults have also been reported^41^. Of note, a recent EEG-fMRI study showed a positive relation between the structural integrity of the cingulate cortex and the amplitude of the NoGo-N2 component during conflict monitoring in younger adults^42^. The here observed effects of prenatal sGC reducing cortical activations in the fronto-parietal network extend early evidence on the link between prenatal sGC exposure and structural brain development^17,18^. Specifically, these results provide new insights into the persisting effects of prenatal sGC exposure on functional mechanisms of cognitive and behavioral control during adolescence.

### sGC exposure affects multiple facets of top-down control

Conflict monitoring entails sub-processes of context representation, conflict detection, action selection and inhibition. In the cued CPT task, stimulus-response conflicts arise when prevalent responses need to be suppressed. Previous computational modeling work showed that attenuated attentional focus on stimulus input can lead to lower N2 amplitudes and longer reaction times^43^. Reduced N2 amplitudes indicate less perceived stimulus-response conflict and were previously suggested to reflect less distinct stimulus-response representations due to difficulties in maintaining attentional focus on stimulus inputs or task contexts^23,44^. Extending these previous findings, the results observed here indicate that prenatal sGC attenuated multiple facets of conflict monitoring. Both the NoGo-N2 amplitudes at the scalp level and the related cortical activations predict individual differences in RTs responding to the target stimuli, with smaller NoGo-N2 amplitudes and cortical activations in the ACC, middle frontal gyrus and SMA predicting longer RTs. Moreover, problems (e.g., lapses) in sustained attention have been characterized by less response consistency in behavior, especially in later task blocks^35–38^. In the present data, although the processing speeds of the prenatally exposed teenagers were not slower than teenagers in the comparison group, their intraindividual RT variability was higher indicating reduced processing consistency^32,34–38^, especially when sustained attention became more in demand at the end of the task. This indicates that prenatal sGC may attenuate sustained attention thereby the processing consistency. Of note, individual differences in the lack of response consistency among the prenatally sGC exposed teenagers can be predicted by individual differences in ACC activations, with greater behavior consistency being associated with greater activations in ACC. Interestingly, a related result at the behavioral level was found in a previous study on effects of prenatal maternal anxiety^38^, which also showed higher intraindividual RT variability towards the end of the CPT task in adolescent boys of mothers who experienced higher levels of anxiety during pregnancy. Considering this previous result together with our new findings suggest that factors influencing prenatal hormonal environment, such as exogenous glucocorticoids as well as maternal anxiety or stress levels, may yield related long-term effects on the development of sustained attention at the behavioral and brain levels. Other than conflict detection which implicates the cingulate regions, we also observed effects of prenatal sGC in attenuating activations in the SMA and the precuneus of the parietal lobe. The SMA is known to implicate action inhibition and selection^45–47^, whereas the precuneus has been related to processes of response conflict adaption^48^ and social-emotional responses^49^. Moreover, recent evidence indicates that hyper functional connectivity between the precuneus and amygdala after stressful events are associated with individual differences in stress responses^39,40^. Given these prior findings, the positive correlation between activation in the precuneus during the CPT task and salivary cortisol reactivity to social stress we found in prenatally sGC exposed teenagers suggest that those teenagers who showed a greater cortisol reactivity to social stress may need to rely more on cortical processes of response conflict adaption during the CPT task and subjectively experienced the task as a more stressful event.

### Strengths and limitations of the study

This study substantially extends previous human research about developmental consequences of prenatal sGC exposure to neurocognitive functions underlying attention and cognitive control during adolescence. With rare exceptions^6,24,25^, existing literature on long-term developmental effects of prenatal sGC exposure potentially could be confounded by other factors associated with preterm delivery (e.g., lower birth weights or other birth outcomes affecting the health status of newborns) that may also influence long-term developmental consequences^26^. By focusing on term-born adolescents, the current study could avoid such confounds. In the past, contradictory effects of prenatal sGC exposure on neuroendocrine development were reported for preterm and term- born neonates, with prenatal sGC downregulated stress reactivity in preterms^50,51^, but amplified cortisol response to stress in term borns^25^. In resolving such inconsistencies, it has been suggested that term-born infants might be even more affected by prenatal sGC exposure as it affects maternal and placental physiology^52^ and the term-born infants are exposed longer to this altered uterine environment^25^. Past results based on this sample showing that prenatal sGC exposure elevated cortisol stress response in children^6^ and the here observed finding of cortisol stress reactivity being sensitive to individual differences in the activation of precuneus in prenatally exposed adolescents are both in line with this interpretation.

The current data could not unequivocally separate effects due to sGC treatments from other potential influences that could be associated with factors such as hospitalization-related maternal stress and other pregnancy complications. Nonetheless, evidence from numerous animal studies applying pharmacological challenges indicate that exogenous sGC yield direct impacts on brain development^13–15^. Furthermore, in a prior study^6^ elevated levels of salivary cortisol response to social stress were observed in children of mothers underwent problematic pregnancy and needed the sGC therapy, but not in those children whose mothers albeit experienced complications during pregnancy and hospital stays but did not receive the sGC treatment. To disentangle the here observed long-term effects of prenatal sGC exposure on neurocognitive development from other potential confounds associated pregnancy complications, future prospective longitudinal research needs to consider additional control groups (e.g., adolescents of mothers who underwent pregnancy complications but did not receive sGC treatment) or clinical randomized controlled trial studies that more systematically investigate the effects of doses or courses of the sGC treatment given to mothers at risk of preterm delivery^9^.

## Conclusion and Outlook

Overexposures to steroid hormones during gestation may disrupt brain and cognitive development in later life periods beyond childhood. Taken together, findings of this study suggest that perturbed levels of steroid hormones in the gestational environment due to exogenous sGC have lasting consequences on adolescent brain development, affecting multiple facets of cognitive and behavioral control. While performing an experimental task requiring conflict monitoring and response inhibition, activations in the fronto-parietal network underlying cognitive flexibility and behavioral control were reduced in term-born adolescents prenatally exposed to sGC. Individual differences in recruiting the anterior cingulate cortex and precuneus in this network are, respectively, associated with variations in response consistency and cortisol stress reactivity.

Keeping the aforementioned limitations of this study in mind, the current results add new insights about persisting influences of prenatal sGC exposure on human functional brain development that last into adolescence. In particular, the observed long-term impacts of prenatal sGC exposure on reducing the consistency of conflict monitoring, along with attenuated functional brain correlates at the scalp and cortical source levels suggest that exogenous influences that lead to increased levels of glucocorticoids during fetal development have lasting impacts on functional adolescent brain development. Given that the effects were observed in the cingulate cortex, precuneus and SMA, prenatal sGC exposure seems to have widespread influences on the maturation of multiple processes important for adaptive cognitive and behavioral control.

These results are of clinical relevance, as about 10% of all pregnancies are at risk of pre-term birth for which prenatal sGC therapy is a standard care, with about 70% to 90% of women who delivered less than 34 weeks received a course of sGC treatment^9,10^. Epigenetic mechanisms during brain development could yield lasting differences in neuroendocrine systems (see^53^ for review). The rapid increase of the level of glucocorticoids towards the end of gestation is accompanied by extensive changes in brain epigenetic profiles^54^ and cortical thinning during adolescence is modulated by glucocorticoid receptor gene expression^55^. Thus, future genomic multimodal imaging studies are necessary in order to gain deeper insights into epigenetic mechanisms that may underlie the persisted long-term effects of prenatal sGC exposure on structural and functional brain development.

## Methods

### Participants

We re-recruited participants from a previous child developmental sample^6,24^. All participants were term-born (>37 weeks of gestation) and were between 14 to 18 years of age (mean = 16) at the time of our data collection. The original sample was recruited in cooperation with the Dresden Neustadt Hospital and the Department of Gynecology and Obstetrics at the unversity hospital (Universitätsklinikum Carl Gustav Carus) in Dresden. Only mothers of healthy term-born infants without receiving pediatric intensive care were contacted. Main exclusion criteria were gestational diabetes, placental insufficiency and regular alcohol, nicotine or drug consumption during pregnancy. The original sample included a comparison group, the PP/sGC group with children born to mothers who underwent pregnancy complications and received sGC treatment, and a third group of children whose mothers although underwent problematic pregnancy but did not receive the sGC treatment. The number of children in the third group was much less than the other two groups, we thus could not re-recruit sufficient number of participants from this subgroup 8 years after the initial study for further control analyses in the current study (other procedural details and criteria for recruiting the original sample can be found in the prior studies^6,24^). For the current study, 34,57% of participants in the PP/sGC group and 28,23% of the comparison group of the original sample could be re-recruited. Ethic approval in accordance with the Helsinki declaration for this study was granted by the TU Dresden ethic committee (EK235062014). Informed consents from both custodians of the teenagers were obtained. All aspects of the experiment were performed in accordance with relevant guidelines and regulations. The final sample consisted of 52 teenagers (see Table 1 for details of sample characteristics). Mothers of adolescents in the PP/sGC group (n = 28) were hospitalized (on average during the 30^th^ week of gestation) due to pregnancy complications associated with risks of preterm delivery (e.g., premature labor pain, vaginal bleeding, cervical insufficiency) and were treated with the standard sGCs therapy. The treatment included one single course of either 2 doses of 12 mg betamethasone given intramuscularly, 24 hours apart or dexamethasone administered as four doses of 6 mg given intramuscularly, 12 hours apart^10^. The comparison group (n = 24) comprised teenagers of mothers without any pregnancy complications, nor were they hospitalized or given the sGC therapy. To assure that our sample size has sufficient power to detect the group and group × condition interaction effects, we conducted power calculations using G*Power (Version 3.1.9.2) based on effect sizes estimated from the prior developmental study^6^, which showed medium effect sizes for the effects of prenatal sGC exposure on stress reactivity and the associated sex interaction. The a-priori power analyses using medium effect size f = 32 (partial *η*^2^ = 0.09) indicated that our sample size (n = 52) yields sufficient power in the range of 0.8 for detecting between-group effects and desirable power in the range >0.95 for detecting group × experimental condition effects.

### Procedure and tasks

About two weeks before the EEG session, basic cognitive abilities and cortisol stress reactivity were assessed in a behavioral session. Markers for verbal ability and perceptual speed were assessed using a modified version of the Spot-a-Word (SaW) task and a computer-based version of the Identical Pictures (IP) task^32^. Cognitive interference susceptibility was measured with the multisource interference task^33^ (MSIT), with interference susceptibility been defined as the ratio of the mean RT during the interference relative to that during the control trials. One teenager did not perform the MSIT task according to task instruction and was not included for the analysis of this measure. After a short break, cortisol reactivity to social stress was measured in response to the Trier Social Stress Test (TSST)^56^, which consisted of a public speaking (5 min) and a mental arithmetic task (5 min). Saliva samples were collected before the onset of the TSST (baseline) as well as 10, 20, 30, and 40 min after test onset. The area under the curve (AUC_i_**)** with respect to salivary cortisol increase during the time course of stress response was calculated for each participant (see 6 for the standard protocol for assessing salivary cortisol levels).

During the EEG session, the participants sat in an electrically and acoustically shielded chamber while performing a variant of the Go-NoGo CPT task used in a prior lifespan study of stimulus-response conflict monitoring^23^. The stimuli consisted of 12 squares (1.07° tall and wide) of different colors that were presented in succession. The participants’ task was to press a response key as fast as possible only when a yellow square (target) was preceded by a blue square (cue). In all other trials, the participants should withhold their responses. Altogether the task consisted of 270 Go pairs (cue followed by target) and 195 NoGo pairs, which were equally divided into 65 prime-based NoGo pairs (cue followed by non-Target), 65 response-based NoGo pairs (non-cue followed by target) and 65 non-cue, non-response NoGo pairs (non-cue followed by non-target). The task was divided into 5 blocks. Stimuli were presented for 200 ms in the center of the screen followed by a black screen. A fixation cross (0.35° tall and wide) was presented 500 ms after the start of each trial for 1650 ms.

### EEG recording

EEG was recorded using Brain Vision Recorder (BrainAmp DC amplifiers, Brain Products GmbH, Gilching, Germany) from 64 active Ag/Al electrodes positioned according to the 10/20 system with a sampling rate of 500 Hz and an online reference placed at the left mastoid. The ground electrode was placed at the forehead. Horizontal and vertical electro-oculograms were recorded by electrodes placed at the outer canthi of the eyes and below the right eye, respectively. Impedances were kept below 10 kΩ.

### Behavioral data analyses

The following performance variables were extracted: percent of three error type, i.e., misses on Go trials, prime based errors (PR-errors) and response based errors (RE-errors) on NoGo trials, mean RT, and intraindividual RT variability indexed by the standard deviation of RTs across Go trials. RTs faster than 100 ms and slower than 2000 ms were excluded from further analyses. To derive an index of sustained attention, intraindividual RT variability were also computed based on trials from the last (5^th^) block of the experiment.

### EEG data preprocessing

EEG data was re-referenced offline to an averaged-mastoid reference using the Brain Vision Analyzer. Further preprocessing of the EEG data was done using the open-source EEGLAB toolbox^57^ and Fieldtrip toolbox^58^ for MATLAB. Data was down sampled to 256 Hz and segmented into epochs of 1500 ms before and 2500 ms after each stimulus. Epochs with severe muscular artifacts were rejected manually first. Independent Component Analysis (ICA) was further applied to the data to detect and remove components of ocular and muscular artifacts. The artifact free data was then bandpass-filtered in the range of 0.5 to 25 Hz and epoched according to the time window of interest from 100 ms before and 1000 ms after Go and prime-based NoGo stimuli, applying a baseline correction 100 ms prior to stimulus onset. We focused on prime-based NoGo pairs only, as they elicited greater response conflict^23^. For further ERP and source localization analyses only epochs with correct responses were considered.

### ERP analysis

ERPs were computed by separately averaging trials for each participant and condition. Peak latencies of the N2 component for Go and NoGo trials were defined as the time at the most negative peak in the individual ERP within a time window of 200 ms to 500 ms. Individual mean amplitudes for the Go- and NoGo-N2 component were obtained by calculating the mean amplitude ± 10 ms around the respective individual peak latency. Group differences in Go- and NoGo-N2 amplitudes at fronto-central channels were analyzed with a linear mixed-effect model, with Group (PP/sGC, comparison group) as the between-subject factor, whereas Condition (Go, NoGo) and Channel (Fz, FCz) as within-subject factors. The channels were chosen based on prior evidence showing that the NoGo-N2 effect is most prominent at fronto-central electrodes^23,27,28^.

### Source localization

To localize neuronal generators of the NoGo-N2 component and test for group effects at the cortical source level, source reconstruction was performed using the sLORETA algorithm^59^ integrated in the Brainstorm toolbox^60^ (http://neuroimage.usc.edu/brainstorm).The current source density was estimated from 60 EEG signal channels in a realistic head model by using the ICBM152 template in Brainstorm. The four different compartments of the head model (scalp, outer and inner skull, cortex) were extracted using the Boundary Element Method^61^ (BEM) that is also implemented in OpenMEEG^62^. A Brainstorm provided default brain surface of 15002 nodes was used for computing the aforementioned compartments. sLORETA computes the current source density distribution for every cortical dipole, resulting in a transition kernel from our 60 averaged scalp EEG signals to 15002 cortical signals. Cortical surface signals within the N2 time window (250 to 280 ms) were averaged and their absolute values were projected to the standard volumetric space in Brainstorm with a spatial resolution of 3 × 3 × 3 mm. The resulted volumetric signals were then imported to SPM8 (http://www.fil.ion.ucl.ac.uk/spm/) for statistical analysis. A full factorial ANOVA was conducted to compare the main effects of Condition (Go vs. NoGo), Group (PP/sGC vs. comparison group) and their interactions with respect to the volumetric activations. For correlational analyses, individual maximum activations of significant clusters were extracted and averaged.

### Statistics

Statistical analyses were performed using the software Package R (version 3.2.3, The R Foundation of Statistical Computing) in RStudio (www.rstudio.com). To compare the main effects of Group (PP/sGC vs. comparison group), Sex (male vs. female), Condition (Go vs. NoGo) and their interactions with respect to the N2 amplitudes, a linear mixed-effect model using maximum-likelihood estimation with participants as a random intercept was conducted using the lme function of the nlme package in R. An initial analysis showed that sex did not reveal any significant main or interaction effects and was thus not considered as a separate factor in all further analyses. In cases of non-normally distributed data, the Mann-Whitney-U test for independent samples was used.

### Ethics

Ethic approval in accordance with the Helsinki declaration for this study was granted by the TU Dresden ethic committee (EK235062014). Informed consents from both custodians of the teenagers were obtained. All aspects of the experiment were performed in accordance with relevant guidelines and regulations.

### Data availability

The anonymous behavioral, EEG and salivary cortisol data will be made available for research purposes upon requests.
